# Use of epoprostenol to treat severe pulmonary vasoconstriction induced by protamine in cardiac surgery

**DOI:** 10.1097/MD.0000000000010908

**Published:** 2018-07-13

**Authors:** Zheng Guan, Xin Shen, Yong-Jian Zhang, Xiao-Gang Li, Yan-Feng Gao, Jing Tan, Hui Yuan, Jing-Jie Liu

**Affiliations:** a Department of Anesthesiology, the First Affiliated Hospital; b Department of Cardiac Surgery, the First Affiliated Hospital; c Department of Neurology, the Second Affiliated Hospital, Xi’an Jiaotong University, Xi’an, China.

**Keywords:** epoprostenol, protamine, pulmonary vasoconstriction

## Abstract

Since there were a few articles to report the treatment of severe pulmonary vasoconstriction induced by protamine in cardiac surgery, we described the use of epoprostenol to reverse this condition.

A total of 5 cases of severe pulmonary vasoconstriction induced by protamine in cardiac surgery were reviewed. The demographic, clinical data and treatment process were obtained. All the patients were followed up.

Severe pulmonary vasoconstriction was occurred 4 to 10 minutes after protamine infusion. The primary sign was sudden hypotension, the pulmonary artery pressure was increased gradually, the arterial oxygen partial pressure was decreased in all the patients. Epoprostenol was infused via pulmonary artery catheter at dosage of 20 to 40 ng/kg·min in all the patients, 2 patients were underwent re-cardiac pulmonary bypass assistance. The hemodynamic instability status lasted 40 to 65 minutes respectively. All the patients were recovered uneventfully.

All physicians should alert to the incidence of severe pulmonary vasoconstriction induced by protamine in cardiac surgery. Use epoprostenol through pulmonary artery catheter could treat pulmonary artery vasoconstriction effectively and safely.

## 1 Introduction

Protamine is mainly used to reverse heparin anticoagulation in cardiac and vascular surgeries. When injected intravenously, the alkaline protamine combines with the acidic heparin to form a neutral salt, thereby eliminating the anticoagulating properties of heparin. The incidence of adverse reaction associated with protamine varies from 1.6% to 10.7%,^[1]^ including allergic reactions, mild transient hypotension, severe pulmonary vasoconstriction, and death.^[2–4]^

Severe pulmonary vasoconstriction induced by protamine is a rare complication, it can cause severe pulmonary artery hypertension (PAH), hypotension, and hypoxemia.^[5]^ Dilating the pulmonary artery and decreasing the pulmonary artery pressure (PAP) is critical treatment of this complication. A few articles described the use of Prostaglandin E1^[6]^ and nitric oxide^[7]^ to decrease PAP in this condition. But most articles gathering this knowledge were case reports. So a systemic research needs to be carried out to disclose the evidence-based knowledge concerning this condition.

Epoprostenol has a potent short-acting vasodilator property, intravenous continuous epoprostenol is used for treatment of PAH, it is effective in idiopathic PAH, and PAH associated with connective tissue disease, portal hypertension or congenital heart disease.^[8]^ Case report showed that Epoprostenol could reverse the severe PAH in patients with β-thalassemia at dosage of 6 ng/kg·min, meanwhile increase cardic output and improve hemodynamic parameters.^[9]^

To the best of our knowledge, no systemic research has been published to discuss the value of epoprostenol in the treatment of severe pulmonary vasoconstriction induced by protamine. To address this relative lack of information, we performed this retrospective analysis.

## 2 Materials and methods

The study protocol was approved by the Ethics Committee of the First Affiliated Hospital of Xi’an Jiaotong University, Xi’an, China. Written informed consent was obtained from all the patients included in this study. From January 2012 to December 2016, a total of 4160 patients were underwent cardiac surgery, all of them were given protamine to reverse heparin anticoagulation in operation, 5 of them were diagnosed as severe pulmonary vasoconstriction induced by protamine, which is defined as an abrupt increase in mean PAP with associated hypotension (SBP ≤90 mmHg) and right ventricular dilation and dysfunction. We retrieved the demographic data, medical history, present illness, clinical presentation, and treatment of the patients. Once severe pulmonary vasoconstriction was confirmed, the epoprostenol was infused via pulmonary artery catheter at a dosage ranging from 20 to 40 ng/kg·min in all the patients, it was adjusted according to the treatment effectiveness, it was suspended when the PAP was declined to normal level. All the patients were followed up.

## 3 Results

### 3.1 Clinical features

The 5 patients were ranged from 22 to 67 years old (Table 1). Case 3 underwent percutaneous coronary angioplasty 5 and 2 years ago because of angina respectively, 3 bare metal stents were implanted in left anterior descending branch, aspirin was taken for 5 years, it is suspended 1 week before this operation. Case 2 had diabetes mellitus (MD) for 8 years, the insulin was used to control the blood glucose.

**Table 1:**
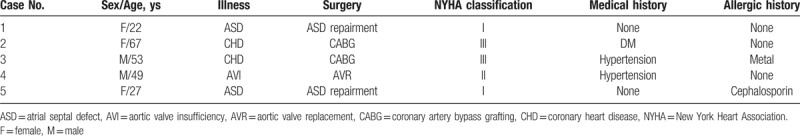
Clinical summary in 5 patients.

### 3.2 The clinical presentation of severe pulmonary vasoconstriction

**Figure 1:**
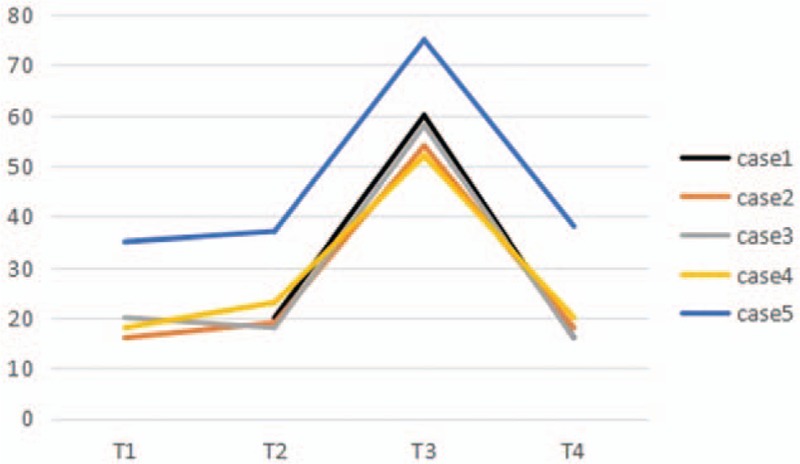
Intraoperative changes of mPAP among the 5 patients. The PAP was not monitored at T_1_ in case 1.

**Figure 2:**
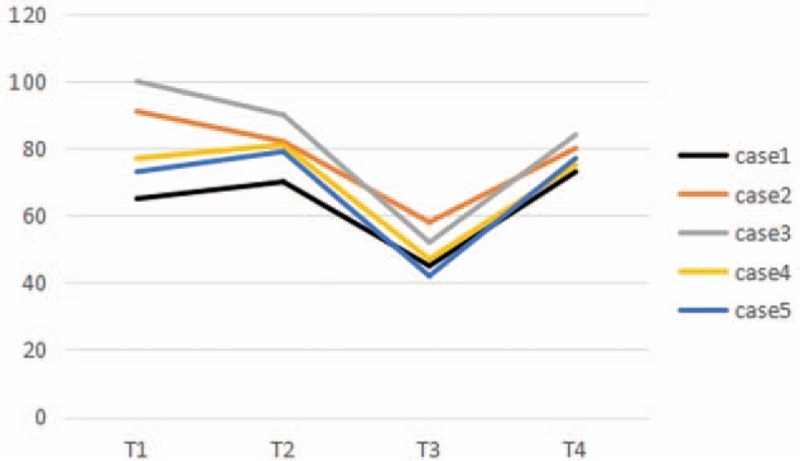
Intraoperative changes of mABP among the 5 patients.

CPB was performed on case 1, 4 and 5, while case 2 and 3 were underwent off-pump surgery. Protamine (3 mg/kg) was infused via aorta (case 1 and 5) and central venous catheter (case 2, 3, and 4) at an infusion rate of 40 mg/min. The arterial blood pressure (ABP) suddenly declined 4 to 10 minutes after protamine infusion, PAP increased gradually in all the patients. The blood gas analysis showed that arterial oxygen partial pressure declined in all the patients, but only case 1 was suffered hyoxemia. Central venous pressure (CVP) increased significantly in case 1, 4, and 5 (the highest CVP was 18, 20, and 19 cmH_2_O, respectively). The peak inspiratory pressure (PIP) increased significantly in case 1 and 5 (the highest PIP was 26 and 25 cmH_2_O, respectively). Severe bradycardia occurred in case 1 and 4. ECG showed that ST-segment depressed in case 3. PAP and ABP were recorded at the following 4 time points in all the patients: T_1_: baseline (postintubation), T_2_: weaning from CPB (case 1, 4 and 5) or preprotamine (case 2 and 3), T_3_: the highest mean PAP (mPAP) and the lowest mean ABP (mABP) postprotamine, and T_4_: at the end of operation. The mPAP and mABP were showed in Figures 1 and 2.

### 3.3 The treatment of severe pulmonary vasoconstriction and follow-up

When the severe pulmonary vasoconstriction was suspected, the fraction of inspired oxygen was increased to 100% immediately; 1 mg of dopamine was injected, followed by intravenous infusion at dosage of 5 to 12 μg/kg·min in all the patients. Epoprostenol was infused via pulmonary artery catheter at dosage of 20 to 40 ng/kg·min in all the patients, it was suspended when the PAP was declined to normal; 0.5 mg of atropine was injected in case 1 and 4 because of severe bradycardia. Epinephrine was infused at dosage of 0.03 to 0.06 μg/kg·min in case 3 and 4 because of severe hypotension. CPB was re-started in case 1 because of severe hypoxemia, in case 5 because of severe PAH, the re-CPB assistance was lasted 40 and 45 minutes, respectively.

The duration of hemodynamic instability status lasted 40 to 65 minutes respectively. The postoperative recovery was uneventful; all the patients were discharged from intensive care unit (ICU) and hospital without any complications. The details were showed in Table 2.

**Table 2:**
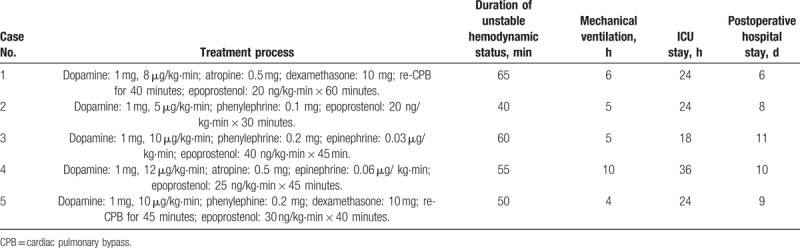
Treatment of severe pulmonary vasoconstriction and follow-up.

## 4 Discussion

Protamine can induce severe pulmonary vasoconstriction in cardiac and vascular surgery. During the past decades, only 5 cases^[5–7,10,11]^ were diagnosed as protamine induced severe pulmonary vasoconstriction (Table 3). The 5 cases we observed were out of a total of 4160 cases from January 2012 to December 2016; the incidence is 1 per 832 (5/4160).

**Table 3:**
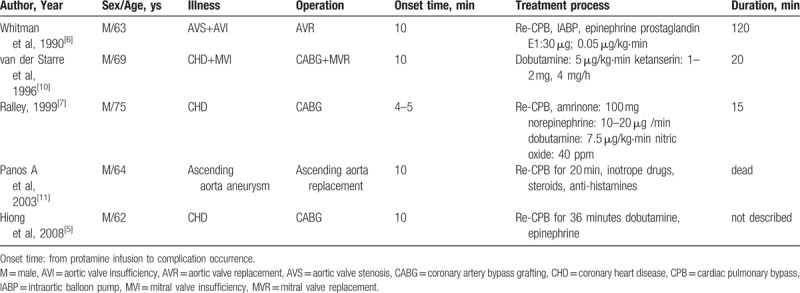
Summary of previously reported severe pulmonary vasoconstriction induced by protamine.

There are 2 mechanisms about pulmonary vasoconstriction induced by protamine infusion. The local excess of heparin-protamine complex, which is caused by rapid infusion of protamine, is thought causing lift-threatened adverse hemodynamic reaction.^[12]^ Besides, animal and human experiments showed that the thromboxane A_2_ is a mediator of this life-threatened adverse hemodynamic reaction,^[13,14]^ the use of aspirin within 1 week before surgery could impair the generation of pulmonary vasoconstrictor thromboxane A_2_, which can decrease the occurrence of pulmonary vasoconstriction.^[14]^

The protamine can be infused via peripheral venous, central venous and aorta, there was minimize change of ABP via aorta, and there was no significant difference of PAP change between the infusion sites.^[14,15]^ DM is risk factor of adverse hemodynamic reaction of protamine following cardiopulmonary bypass.^[16]^ In DM patients who were treated with insulin, there was delayed-type hypersensitivity to protamine.^[17]^ Previous studies showed that the allergic shock and death^[18]^ and anaphylactoid reaction to protamine^[19]^ during open heart surgery were both occurred in DM patients. So adverse hemodynamic reaction to protamine should be alerted in DM patients.

Maintaining normal cardiac function, increasing ABP, decreasing PAP are the main strategies to treat pulmonary vasoconstriction induced by protamine. Vasoactive agents, cardiotonic agents and heart-assist device such as CPB and intra-aortic balloon pump (IABP)^[6]^ can be used to maintain normal cardiac function and ABP. In our study, dopamine and epinephrine were used to maintain ABP, CPB was restarted in two patients. Epoprostenol,^[8]^ protacyclin, and prostaglandin E1 are all prostaglandins, previous studies showed that prostacyclin inhalation^[18]^ and prostaglandin E1 infusion^[6]^ can decrease PAP effectively, but there is a few article to report the use of epoprostenol in decrease the PAP and reverse the pulmonary vasoconstriction induced by protamine. Because of the potent short-acting vasodilator property of epoprostenol, it is suitable used during operation and emergency situation, we used epoprostenol via pulmonary artery catheter to dilate pulmonary artery and decrease PAP, the PAP declined to normal in 30 to 60 minutes, while the peripheric vascular resistant and ABP decreased slightly. Though epoprostenol can inhibit platelet aggregation by increasing cAMP in platelets,^[20]^ the postoperative bleeding was not increased in all the patients.

Pulmonary vasoconstriction induced by protamine was reversible, so there were not persistent complications, such as ischemic heart disease, ischemic cerebrovascular disease, liver, or renal dysfunction. In our study, all patients were recovered uneventful because of the timely treatment of the protamine induced severe pulmonary vasoconstriction.

This study had the following limitations. First, the small number of cases included in our study made it difficult to reach definite conclusion. Second, this was a retrospective study, so uncontrolled bias may be inevitable. A large patient sample and a perspective study were needed to gain a definite conclusion.

## 5 Conclusion

All physicians should alert to severe pulmonary vasoconstriction induced by protamine in cardiac surgery. The primary symptom was hypotension shortly after protamine infusion, combined with increasing of PAP. The use of epoprostenol via pulmonary artery catheter can decline PAP and reverse this complication effectively and safely.

## Author contributions

**Conceptualization:** Zheng Guan, Jing-Jie Liu.

**Data curation:** Yong-Jian Zhang, Xiao-Gang Li, Jing Tan, Hui Yuan.

**Formal analysis:** Xiao-Gang Li, Yan-Feng Gao, Jing Tan.

**Investigation:** Yan-Feng Gao.

**Methodology:** Hui Yuan.

**Project administration:** Zheng Guan, Xin Shen.

**Resources:** Yong-Jian Zhang.

**Supervision:** Jing-Jie Liu.

**Writing – original draft:** Zheng Guan.

**Writing – review & editing:** Jing-Jie Liu.
